# Polybenzoxazine Resins with Cellulose Phosphide: Preparation, Flame Retardancy and Mechanisms

**DOI:** 10.3390/polym13244288

**Published:** 2021-12-07

**Authors:** Hui Li, Zhangmei Sun, Chunxia Zhao, Yuntao Li, Dong Xiang, Yuanpeng Wu, Jixuan Wei, Yusheng Que

**Affiliations:** 1 School of New Energy and Materials, Southwest Petroleum University, Chengdu 610500, Sichuan, China; Aa2515954054@163.com (Z.S.); yuntaoli@swpu.edu.cn (Y.L.); d.xiang@swpu.edu.cn (D.X.); wuyp362@126.com (Y.W.); wjx15076120896@163.com (J.W.); m15351372549@163.com (Y.Q.); 2 State Key Laboratory Oil and Gas Reservoir Geology and Exploitation, School of New Energy and Materials, Southwest Petroleum University, Chengdu 610500, Sichuan, China; 3 The Center of Functional Materials for Working Fluids of Oil and Gas Field, School of New Energy and Materials, Southwest Petroleum University, Chengdu 610500, Sichuan, China

**Keywords:** thermosets, flame retardant, phosphate cellulose, thermal properties

## Abstract

Phosphated cellulose (PCF) was synthesized based on urea, phosphated acid and cellulose. The structure of the PCF was confirmed by Fourier transform infrared (FTIR) spectroscopy and scanning electron microscopy coupled with the Energy Dispersive Spectrometer (SEM-EDS). Benzoxazine (Ba)/PCF hybrid materials were fabricated and thermally cured to prepare polybenzoxazine composites (PBa/PCF). The effects of PCF on the curing temperature of Ba were analyzed through differential scanning calorimetry (DSC). The thermogravimetric (TGA) results demonstrated an increased char residue of 50% for the PBa composites incorporating PCF-5% compared with the pure PBa. The peak heat release rate (PHRR) and total heat release (THR) values of the PBa/PCF-5% composites clearly decreased by 58.1% and 16.5% compared to those of the pristine PBa. The smoke released from the PBa/PCF system significantly reduced with the loading of PCF. Moreover, the limited oxygen index (LOI) and vertical burning test level (UL-94) of PBa/PCF-5% reached up to 31 and V0. The flame retardant mechanism of the PCF in the PBa matrix was investigated TG-FTIR and char residues analysis. Finally, the dynamical mechanical analysis (DMA) results demonstrated that the Tg of the PBa/PCF composites was approximately 230 °C, which does not affect further applications of PBa composites.

## 1. Introduction

Polybenzoxazine (PBa) is a new type phenolic resin, which can be synthesized by formaldehyde, primary amines and phenols through Mannch reaction [1,2,3]. PBa offers outstanding performance in comparison to traditional phenolic resin, such as its flexible molecular design, near-zero shrinkage behavior during polymerization, low water absorption, low dielectric constant, low coefficient of thermal expansion and so on [4,5,6,7,8]. These characteristics make it possible to broaden the application prospects of PBa beyond aerospace, electronics and automobile manufacturing [9,10,11,12,13]. However, PBas, especially those that are bisphenol-A-based, are easy to flame with a limited oxygen index (LOI) of about 21, which makes them undesirable for many engineering applications. Various kinds of flame retardants and fire-quenching strategies have been developed to reduce the high combustibility of PBa. The intrinsic chemical flame retarding method involves introducing flame retardancy elements into the PBa molecular chain based on the flexible molecular design of benzoxazine (Ba) monomers. Zhu et al. synthesized novel Ba monomers from P-nitrophenol, paraformaldehyde and aniline [14]. The nitro group promoted the ring-opening polymerization of Ba monomers, as well as favoring higher thermal stability and char residue of PBa compared to the phenol-aniline-based Ba monomers and polymers, respectively. Lin et al. synthesized a novel flame retardant PBa copolymer with phosphorus-containing Ba monomers [15]. The glass transition temperature (T_g_), thermal decomposition temperature (T_d_) and flame retardancy of the PBa were all clearly enhanced. Halogen-free flame-retardant additives have recently attracted much attention from both researchers and industrialists due to their environment-friendly, convenience, low cost and high efficiency [16,17]. Demir et al. prepared a new class of polybenzoxazine/montmorillonite (PBa/MMT) nanocomposites and the char yield increased from 16% to 35% with 10% of MMT [18]. PBa composites based on α-zirconium phosphate (α-ZrP) demonstrated improved thermal stability and char residue formation [19]. Amongst all halogen-free flame-retardant additives, phosphorous-containing compounds are popular non-halogen additives, known to be effective at improving the flame retardancy of polymers through their ability to promote char layer formation. The PBa composite exhibited a 62.5% reduction in peak values of heat release (PHRR)and a 49.3% decrease in total heat release (THR) with the incorporation of 5% polyphosphazene microspheres [20]. Meanwhile, epoxy resin (EP) cured by a phosphorus-containing hardener mixture exhibited significantly reduced THR and total smoke production [21]. In Hong et al.’s work [22], a novel piperazine-containing additive with phosphorus and alkyny (PPBPP) was used to flame-retard thermo-plastics polyurethane (TPU). They found that 3.0 wt% PPBPP endowed TPU with a UL-94 V-0 rating. Phosphorus-based flame retardants can produce phosphoric acid, polyphosphoric acid during heating. These acid products promote the dehydration and carbonization of substrate to form the char layer, which prevents the transfer of oxygen, heat and mass between the burning zone and the inner, unburnt area [23,24,25]. In addition, phosphorus-containing radicals produced by the decomposition of phosphorous flame retardant can capture radicals in the gas phase and terminate chain reaction, as well as reducing the further decomposition of polymer materials [26,27]. With the current focus on the sustainable development of society, renewable bio-based flame retardant agents have gained extensive attention. Currently, bio-based materials, such as amino trimethylene phosphonic acid (ATMP), starch, lignin, β-cyclodextrin and chitosan (CS) are used as eco-friendly additives to improve the flame retardancy of polymers [28,29,30,31]. Thirukumaran et al. prepared a new class of modified chitosan-based benzoxazine precursor/amino cellulose ((E-ch/AC) film [32]. The mechanical performance, thermal properties and char yield of the polymer films all improved.

Inspired by the above published reports, phosphated cellulose (PCF) was synthesized and used to improve the flame retardancy of PBa in this work. The effects of PCF on the thermal stability, flame retardancy, burning behavior and mechanical properties of PBa were measured and discussed. The flame retardant mechanism of the composite was investigated by analyzing char residues and gas phase products.

## 2. Experimental Section

### 2.1. Materials

Bisphenol A-based benzoxazine (Ba) was provided by Sichuan Tiance Jucai Technology Co., Ltd. (Chengdu, China). The 98% phosphite, 99.5% urea, ethanol, and acetone were purchased from Chengdu Kelong Chemical Reagent Factory (Chengdu, China). The 50 μm cellulose was supplied by Innochem (Beijing, China). All the chemicals were used as received without further purification.

### 2.2. Phosphorylation of α-Cellulose

**Figure 1:**
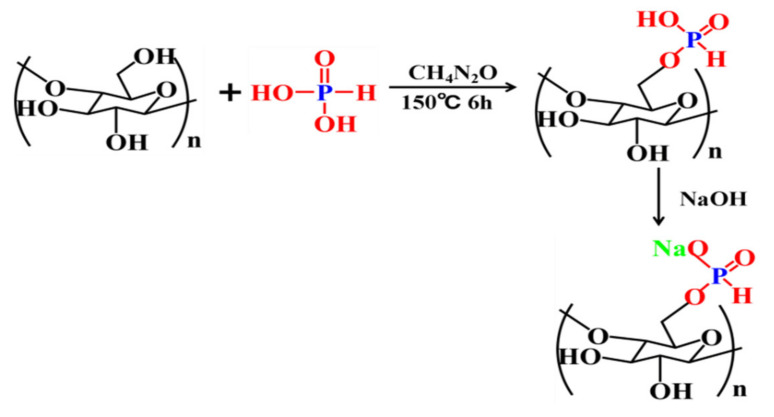
The phosphating process of cellulose.

Phosphated cellulose (PCF) was synthesized according to previous research [33]. To begin with, 12.48 g (0.208 mol) urea was added into a 250 mL three-neck bottle and heated to 140 °C in a N_2_ atmosphere until the urea was dissolved completely. Next, 2.00 g (0.0123 mol) cellulose and 10.28 g (0.1253 mol) phosphorous acid were added alternately to the molten urea. The reaction was carried out at 150 °C for 6 h. The reacted mixture was dissolved in 250 mL 1 N aqueous sodium hydroxide and precipitated with ethanol. This process was repeated three times, in order to remove the urea and the unreacted phosphite. The modified cellulose was freeze dried. The final phosphorylated cellulose was light yellow and water-soluble. The phosphating process of the cellulose is shown in Figure 1.

### 2.3. Preparation of PBa/PCF Composites

**Figure 2:**
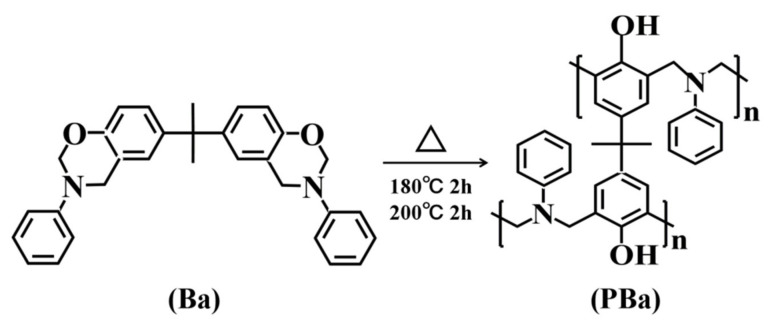
The chemical structure and curing reaction of Ba.

First of all, 0.9 g PCF and 90 g Ba were distributed in 30 mL acetone in a 250 mL three-necked flask under ultrasound at room temperature. Next, the acetone was removed by oil-bath heating, aided by a rotary evaporator. Finally, the mixture was poured into a glass, mold treated with release agent and preheated for curing at 180 °C/2 h, followed by 200 °C/2 h. The cured product was labeled as PBa/PCF-1%. The pure PBa, PBa/PCF-3% and PBa/PCF-5% were produced in a similar process. The chemical structure and curing reaction of Ba are shown in Figure 2.

### 2.4. Characterization

The surface functional group of pulverized phosphatized cellulose powder was tested through Fourier transform infrared (FTIR) spectroscopy using a Nicolet 6700 spectrometer (Thermo Electric Corporation, West Chester, PA, USA) in the range of 4000–500 cm^−1^. The morphology of the samples was analyzed through the scanning electron microscope (SEM) platform, using a ZEISS EV0 MA15 SEM from Carl Zeiss Micro Image Co., Ltd. (Jena, Germany) The elemental analysis of the sample was implemented in EDS using a ZEISS EV0 MA15 from Carl Zeiss Micro Image Co., Ltd. The thermal stabilities were measured on a thermogravimetric analyzer (DSC823 TGA/SDTA85^e^) in a nitrogen and air atmosphere with a heating rate of 10 °C/min, from 40 °C to 800 °C. The curing temperature of the Ba and Ba/PCF mixtures was measured using non-isothermal DSC Q20 (TA Instruments, New Castle, DE, USA) at a heating rate of 10 °C/min from 40 °C to 300 °C. The fire performance of the materials was analyzed using a Cone calorimetry (CONE) test by ASTME1354/ISO 5660. The size of the samples was 100 × 100 × 3.2 mm^3^. The samples were enclosed in aluminum foil and measured at 35 kW/m^2^ heat flow conditions. A dynamic mechanical analysis (DMA) was carried out using a Q800 analyzer (TA Instruments) from 40 to 300 °C, at a heating rate of 5 °C/step, with a frequency of 1 Hz in air. Samples with dimensions of 40 × 10 × 3.2 mm^3^ were used. A thermogravimetric–Fourier transform infrared (TG-FTIR) analysis was performed using a Perkin-Elmer STA 6000 (Waltham, MA, USA) in a nitrogen and air atmosphere, with a heating rate of 10 °C/min from 40 °C to 800 °C. The limiting oxygen index (LOI) values were tested using an HC-2C Oxygen Index Flammability Gauge (Jiangning, China), according to ASTM D2863-97. The dimensions of all the samples were 130 × 3.5 × 3.2 mm^3^. The vertical burning test (UL-94) was carried out on a CZF-2 instrument (Jiangning, China), according to GT/T 8333-2008. The dimensions of all the samples were 125 × 12.7 × 3.2 mm^3^. The structures of the char residues obtained from the CONE test were obtained through Raman spectroscopy, using a ID Raman micro IM-52 from Ocean Optics (Dunedin, FL, USA) fitted with a laser, at a wavelength of 785 nm.

## 3. Results and Discussion

### 3.1. Characterization of PCF

**Figure 3:**
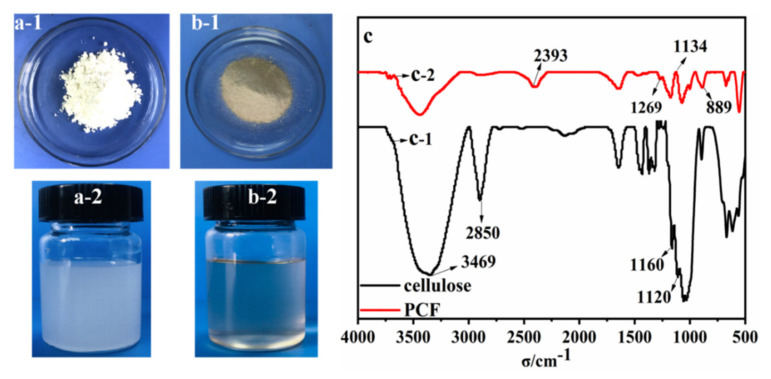
Comparison of the morphology and solubility in water of cellulose (**a**-**1**,**a**-**2**) and PCF (**b**-**1**,**b**-**2**); FITR of cellulose (**c**-**1**) and PCF (**c**-**2**).

The cellulose took the form of a white-like powder and was not insoluble in water, as shown in Figure 3(a1,a2), respectively. Clearly, the PCF changed to faint yellow and dissolved in water to form a transparent solution (Figure 3(b1,3b2)), which preliminarily demonstrated the phosphating reaction of the cellulose. Cellulose macromolecule belongs to polysaccharide, which is composed of d-glucose with β-1, 4 glycosidic bonds [34]. The abundant hydroxyl groups in cellulose make it easy to be modified. It has been reported that phosphoric acid could react with the hydroxyl group on the C-6 atom in cellulose [35,36,37,38]. The FTIR spectra of cellulose and PCF are presented in Figure 3(c1,c2). The characteristic absorption peaks of cellulose at 1160 cm^−1^ and 1120 cm^−1^ belong to the vibration of the –C–O–C- in the glucoside unit or β-(1–4) glucosinic bonds. The peaks from 2850–3000 cm^−1^ were attributed to the –C–H stretching vibration of the –CH_2_ groups. The characteristic absorption peak of 3469 cm^−1^ corresponds to the vibration for the –OH groups. The intensity of the –OH stretching of PCF clearly weakened, proving that the –OH group on the C-6 atom in α-Cellulose was consumed during the phosphating reaction. New peaks appeared at the PCF FTIR spectrum (Figure 3(c2)): the characteristic absorption peak at 2393 cm^−1^ corresponded to the P–H absorption, the peak at 889 cm^−1^ was due to the P–O–C absorption and the peaks at 1269 cm^−1^ were attributed to the –P–O– absorption. Furthermore, the characteristic peak at 1134 cm^−1^ was ascribed to the absorption of –P=O in PCF. The above phenomena and discussion confirm the successful phosphating reaction of the cellulose.

### 3.2. Morphology of Cellulose and PCF

**Figure 4:**
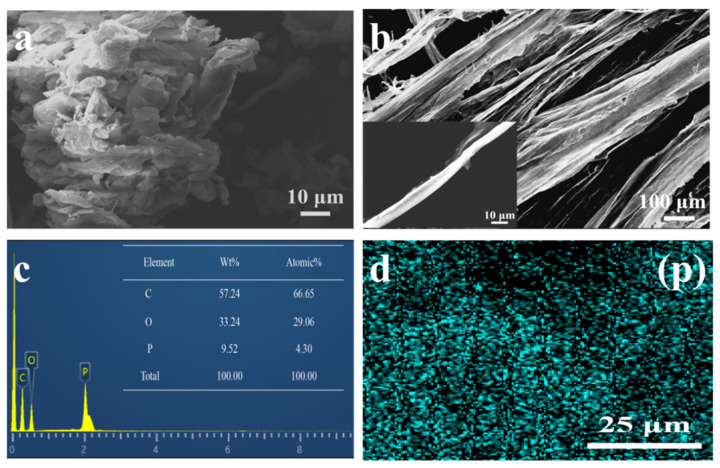
SEM images of cellulose (**a**) and PCF (**b**); (**c**) EDS spectra of the PCF; (**d**) the PCF with elemental mapping images of P.

The morphologies of the cellulose (Figure 4a) and PCF (Figure 4b) were investigated by SEM. Apparently, cellulose without phosphorylation is in a discrete state and features a smooth surface. However, PCF exhibits clear fibrous tissue with a rough surface, which can be obtained by freeze drying. An EDS test was performed to detect the presence of P element with a PCF content of about 9.5%. The uniform distribution of the P elements can be seen in Figure 4d, marked as blue signals. The analysis of FTIR and SEM-EDS proved the successful preparation of the PCF in this study.

### 3.3. Curing Temperature of Ba and Ba/PCF Systems

**Figure 5:**
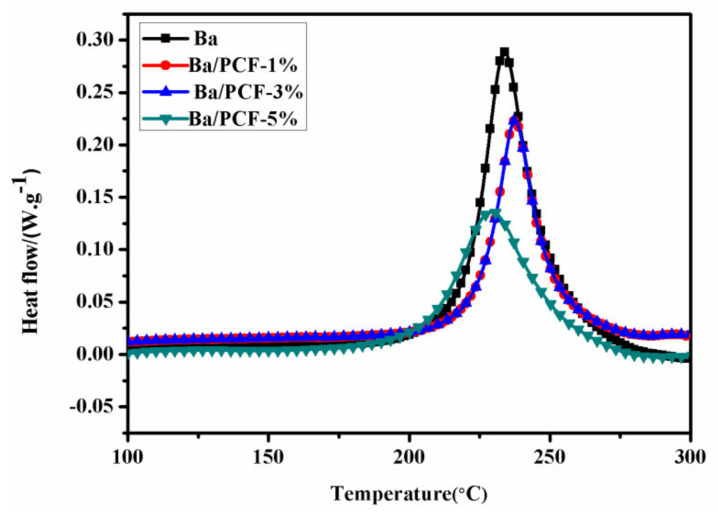
DSC curves of the Ba and Ba/PCF systems at a heating rate of 10 °C/min.

The Curing temperatures of the Ba and Ba/PCF systems were analyzed by the non-isothermal DSC at a heating rate of 10 °C min^−1^, as shown in Figure 5. The curing characteristic parameters, including the initial curing temperature (T_i_), curing peak temperature (T_p_) and final curing temperature (T_f_) are listed in Table 1. A single exothermic peak for the ring-opening thermal curing of the Ba and Ba/PCF systems was observed. However, the T_i_ of Ba decreased with the addition of PCF. Specifically, the T_i_, T_p_, and T_f_ of Ba with 5% PCF decreased by 29 °C, 5 °C, and 12 °C respectively, compared with those of the pure Ba. This phenomenon may be ascribed to the catalytic effect of PCF on Ba. This was mainly attributed to the P of PCF. The element P with empty electron orbits around the nuclei could accept the electron pairs of the O atom on the oxazine ring to form coordinate bonds when the PCF was mixed with the Ba. Once the P atom coordinated with the O atom, the –CH_2_–O bond is prone to fragility because of the electron with the drawing nature of P [39]. Next, cleavage occurs to form the intermediate of iminium-ion/carbenium-ion resonance structures. The electron density of this position can be lowered by the coordination of P with the O atom [40]. The resulting phenolic hydroxyl group further catalyzes the curing reaction of Ba. Hydroxyls of PCF could interact with oxygen and nitrogen atoms in Ba monomers and resulted in the formation of inter-molecular hydrogen bonds. With the temperature increasing, hydrogen bonds broke and released free hydroxyl to accelerate the ring-opening reaction of the oxazine ring [41]. The curing temperature was set as 180 °C/2 h, followed by 200 °C/2 h, to fully cure the Ba monomers and Ba/PCF mixture.

### 3.4. Thermal Stability

**Figure 6:**
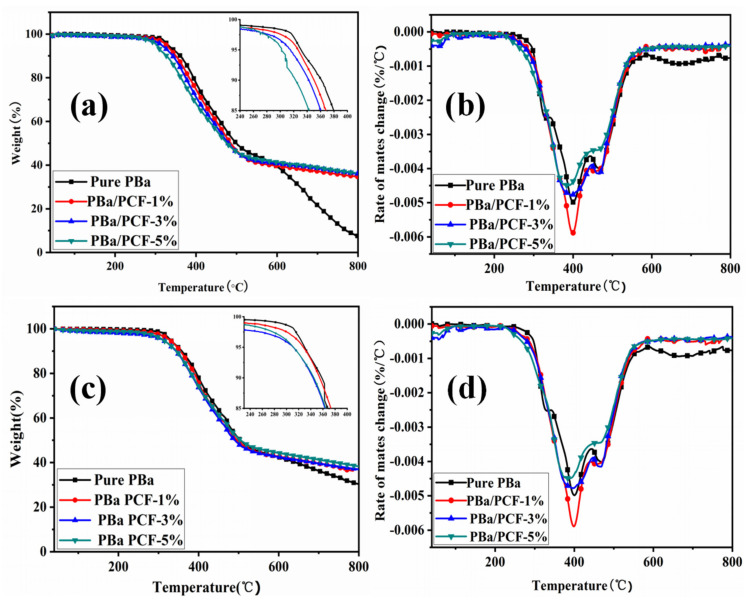
TGA (**a**) and DTG (**b**) curves of PBa and PBa/PCF composites in air and N_2_ atmospheres (**c**,**d**).

The thermal stability of the PBa and its composites was detected through TGA and DTG (Figure 6). The onset decomposition temperature (T_onset_) expressing 5% mass loss, the temperature at the maximum mass loss rate (T_max_) and amount of char residues are collected from Figure 6a–d, which are shown in Table 2. Both the T_onset_ and T_max_ lowered significantly with the addition of PCF, as shown in Figure 6 and Table 2. The T_onset_ reduced from 331 °C for pure PBa to 326 °C for PBa/PCF-1% in air, and from 334 °C to 323 °C in N_2_. The T_onset_ and T_max_ of the PBa/PCF-5% composite reduced by 29 °C and 30 °C in an air atmosphere and by 18 °C and 39 °C in N_2_, compared with than of the pure PBa. The char residue of PBa was 26% at 700 °C, and reduced to 7% and 800 °C in air. Interestingly, the char residue’s content was enhanced with the incorporation of PCF in the PBa matrix, especially at high temperatures. The char residue of PBa/PCF-1% reached up to 37% at 700 °C and 34% at 800 °C in air. A slight increase in the char residue’s content was observed along with the increase in the incorporation of PCF from 1% to 5% in the PBa, both in air and N_2_. The chair residue’s enhancement was higher in air than in N_2_ above 700 °C, which implied that PCF effectively promoted char formation during the combustion of PBa, especially in air. On the whole, the thermal decomposition reaction in air exhibited almost the same tendency of PBa/PCF composites as that in N_2_.

There were two main patterns of thermal oxidative degradation for PBa and its composites. The thermal decompositions all primarily appeared in the range of 300–500 °C. The first step was shown in 300–420 °C. This is mostly because of the degradation of phenols and amines in the PBa matrix during combustion. Less-stable O=P–O bonds in PCF lead to lower T_initial_ in PBa/PCF composites. Groups containing phosphorus decompose to react with PBa during heating, which can accelerate the degradation of PBa composite to form a stable char layer [42]. In addition, phosphoric acid, polymetaphosphoric acid, H_2_O gas and so on can be produced by PCF above 300 °C [43], which can help stable char residue to resist the heat and substance transfer, thus preventing further decomposition. The second stage is 420–500 °C, which mainly corresponds to a further thermal degradation and crosslinking reaction among phosphate groups and the main chain of PBa molecule, resulting char layers. These char layers on the surface of the matrix are able to generate an effective physical barrier to retard the transfer of gases, heat and interior thermal decomposition products. Hence, they prevent combustion.

### 3.5. Flammability Performance of PBa and Its Composites

UL-94 vertical burning testing and LOI were used to measure the influence of PCF on the flame retardancy of PBa. The LOI value was 21 for pure PBa, which increased to 24, 28 and 31 with the loading of PCF 1%, 3% and 5% in the PBa matrix, respectively. In addition, the UL-94 level improved from the NO level to the V0 level. The results of the UL-94 vertical burning and LOI testing demonstrate the contribution of PCF in PBa to the flame retardant properties.

**Figure 7:**
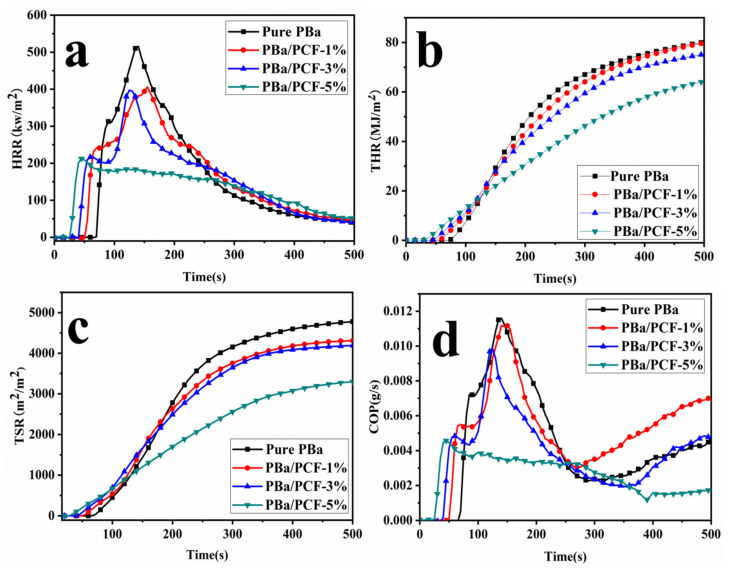
HRR (**a**), THR (**b**), TSR (**c**), and COP (**d**) versus time curves of PBa and PBa/PCF composites obtained from CONE test.

Cone calorimeter (CONE) is a fire testing instrument that can assess the flammability characteristics and potential fire safety of polymers. CONE tests were used to evaluate the properties of the PBa composites when burning. The heat release rate (HRR), total heat release (THR), carbon monoxide production (COP) and total smoke release (TSR) are shown in Figure 7. The time-to-ignition (TTI), peak of HRR (PHRR), average heat release rate (AvHRR) and mass loss rate are presented in Table 3.

Time-to-ignition (TTI) is used to test the effect of PCF on ignitability. It can be observed in Table 3 that the TTI value of the original PBa was longer than that of the PBa composites. Specially, the TTI value of PBa/PCF-5% was only 23 s, which was much shorter than the value of 66 s obtained for the pure PBa. This may have been because the instability of P–O and P=O of the PCF could shorten the TTI [44,45,46], which promotes the formation of the cross-linking structure in PBa composites, decreases the decomposition of PBa and improves the flame retardancy of the matrix. The AvHRR of composites decreases significantly with the incorporation of PCF (Table 3). The PBa/PCF-5% displayed an AvHRR of 110.3 kW/m^2^, which was 30.3% lower than that of the pure PBa. Meanwhile, the mass loss rate of the PBa/PCF-5% reduced from 74.5% to 65.9%, compared with original PBa. The combination of the lower value of AvHRR and the mass loss rate demonstrate that PBa composites could partially undergo a process of char formation rather than burning.

In Figure 7a and Table 3, it can be observed that the PHRR values for the PBa/PCF-5% notably decreased. The PHRR was 212.3 kW/m^2^ for the PBa/PCF-5%, with a reduction of 58.8% compared with the PBa. These phenomena indicated the superior fire performance of composites. The distinct reduction in PHRR was mainly due to the cross-linking char formation process promoted by the introduction of PCF in the PBa. Figure 7b exhibits the THR curves for the PBa/PCF composites. A significant finding is that the value of THR for pure PBa decreased from 83.9 to 70.1 MJ/m^2^ compared with the PBa/PCF-5%. The cross-linked char layer formed during the carbonization process of the PBa/PCF composites can produce a physical barrier, which contributes to a reduction in matter transformation during the burning process and in the risk of fire. As is well known, smoke causes more deaths than burning. Meanwhile, the production of soot mainly resulted in incomplete combustion during the burning of the polymers. The decrease in TSR (Figure 7c) implies that the loading of PCF has a significant smoke-suppressing effect in PBa composites, resulting in a decreased risk of asphyxiation in a fire disaster. The value of COP can be used to assess the toxicity of materials in combustion. The loading of PCF, especially with 5% content, clearly reduced the COP value in comparison with the original PBa (Figure 7d). The loading of PCF can significantly decrease the amount of CO and reduce toxicity during the combustion process of PBa/PCF composites. Consequently the prepared PBa/PCF composites can save time for safe evacuation during a fire.

### 3.6. Analysis of Flame Retardancy Mechanism

#### 3.6.1. Condensed Phase Analysis

**Figure 8:**
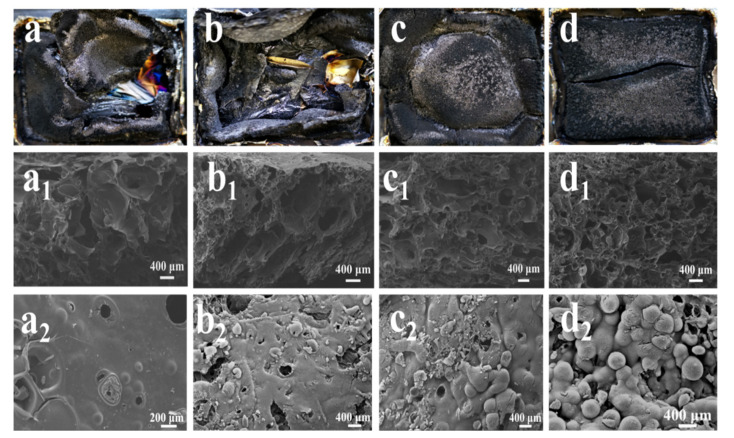
Digital photographs of the char layers (**a**–**d**) and SEM images of the residual char interior regions (**a_1_**–**d_1_**) and outside surfaces (**a_2_**–**d_2_**) of (**a**) pure PBa, (**b**) PBa/PCF-1%, (**c**) PBa/PCF-3% and (**d**) PBa/PCF-5%.

The analysis of residual chars could provide an insight into flame resistant properties and further reveal the possible mechanisms of char formation for flame retardant materials [47]. High-quality char can act as a good insulating barrier to greatly limit the diffusion of volatiles into the flame zone. The morphology of the residual char of the pure PBa and PBa composites obtained from the CONE tests was observed through SEM, as shown in Figure 8. The char layers of the pure PBa and the PBa/PCF-1% were broken and cracked, as demonstrated in Figure 8a,b. However, the PBa/PCF-3% and PBa/PCF-5% exhibited continuous, compact and tight char surfaces, as demonstrated in Figure 8c,d. These phenomena fully prove that a higher incorporation of PCF can improve the quality of the char layer more effectively during burning. SEM images of the outside surface and inner structure of the pristine PBa and PBa/PCF char layers are presented in Figure 8(a1–d2). The residual chars of the pure PBa were poor in flexibility and prone to destruction; therefore, they cannot inhibit CO and CO_2_ gases or protect the PBa matrix. Nevertheless, as shown in Figure 8(b1–d1), with the loading of PCF, the inner structures of the composites’ charred layers became more consecutive, uniform, bumpy and porous, with many smaller hollow cells. Meanwhile, the PBa/PCF-5% exhibited the most continuous and compact outer surface, as shown in Figure 8(a2,d2). This suggests that PCF is the key factor in the formation of high-quality intumescent char layers. Generally speaking, intumescent charred layers help to slow down the exchange of heat and mass between the gas and solid phases, which can offer a better flame shield for underlying polymers during degradation.

As is well known, phosphorus-based flame retardants can accelerate the degradation reaction of polymer materials [44,45,46]. The PCF in PBa matrix underwent thermal degradation during the initial decomposing stage to form polyphosphoric acid, which can be cross-link-reacted with PBa during burning. The dehydration reaction depends on the hydroxyl groups in PBa, which can form a cross-linked network structure during the preliminary decomposition. Next, the firm and intumescent charred layer is formed to protect the polymer’s interior significantly. Furthermore, intumescent flame retardants exert a synergistic flame-retardant effect between the acid, gas, and charring source. In this study, the acid source was PCF, and PBa was the gas and charring source [43]. In the process of combustion, the acid source effectively improved the carbonization of the PBa, which formed a compact and continuous charring layer and hindered the thermal decomposition of the PBa, resulting in the prohibition of the mass and heat transfer. The oxygen concentration was diluted by the generated noncombustible gas, which improved the flame-retardant’s performance. When the amount of oxygen and heat in the environment is insufficient, it is difficult for the material to burn, which leads to self-extinguishment [48].

**Figure 9:**
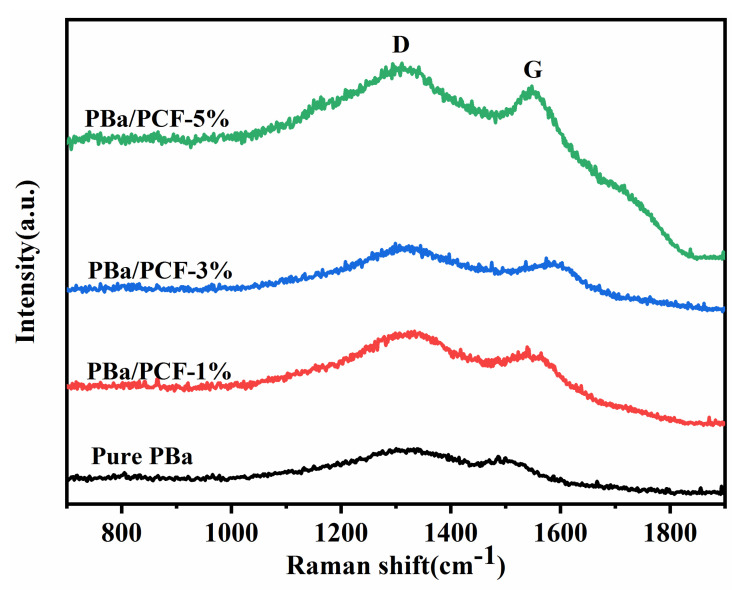
Raman spectra of char residues for samples collected from CONE test.

Raman spectroscopy was employed to investigate the structure of the char residues obtained from the CONE test. The char residues exhibited two characteristic bands, as presented in Figure 9. The D band at 1315 cm^−1^ and the G band at 1550 cm^−1^ contributed the vibration of amorphous carbon and the vibration of crystalline graphite carbon, respectively. In general, the integral area ratio (I_D_/I_G_) of the two bands was dependent on the graphitization degree of the carbonaceous materials. The lower I_D_/I_G_ value demonstrated a higher graphitization degree. It can be inferred that the I_D_/I_G_ values of the pure PBa and PBa/PCF nanocomposites were 1.023, 1.023, 1.018 and 1.016, respectively. Clearly, with the addition of PCF, the I_D_/I_G_ values reduced. This phenomenon implies that the char layers of PBa/PCF composites were more compact compared with the original PBa. The results and the discussion of the Raman spectroscopy were in good agreement with those of the SEM.

#### 3.6.2. Gas Phase Analysis

**Figure 10:**
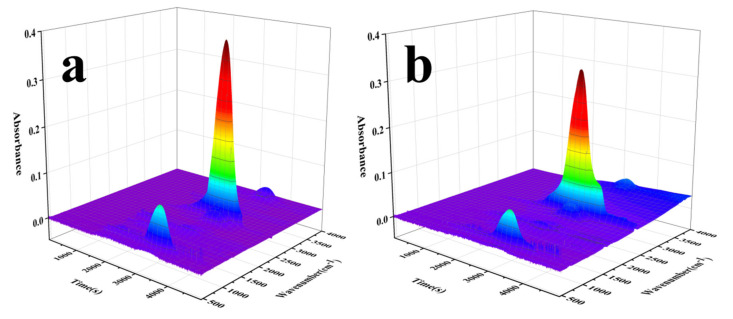
3D TG-FTIR images of pyrolysis products of (**a**) Pure PBa and (**b**) PBa/PCF-5%.

**Figure 11:**
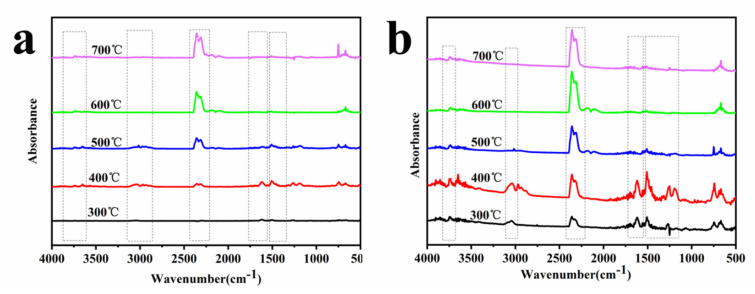
FTIR spectra of (**a**) pure PBa and (**b**) PBa/PCF-5% at different temperatures.

In order to directly distinguish the changes in the peak intensities of the main pyrolysis products of PBa and its composites at various temperatures, TG-FTIR tests were carried out. The results are described in Figure 10 and Figure 11. The main pyrolysis products of pure PBa during thermal decomposition were water and amine-containing compounds (3500–3800 cm^−1^), compounds containing alkane (2900–3000 cm^−1^), CO_2_ (2350 cm^−1^), and aromatic compounds (1500–1750 cm^−1^). The loading of PCF contributed an early release of water, amine-containing compounds, hydrocarbons, CO_2_ and the aromatic compounds compared with pure PBa. This phenomenon revealed that the addition of PCF played a significant part in catalyzing the thermal decomposition of the PBa. To compare precisely, the FTIR spectra of the gas phase for pure the PBa and PBa/PCF-5% at various temperatures are presented in Figure 11. The characteristic bands for the common gaseous products of PBa/PCF-5% were mostly same as those of the pristine PBa. PBa/PCF-5% exhibited a new, strengthened peak at around 1269 cm^−1^, which was due to the characteristic adsorption of the –P–O– bond. This implied that some of the phosphorous groups in the PBa/PCF composites degraded thermally into the gaseous phase. Free radicals could effectively capture or terminate in the gas phase, so that the process of heating or combustion was delayed. Finally, PCF plays the role of flame retardant in PBa/PCF composites.

### 3.7. Dynamic Mechanical Properties

**Figure 12:**
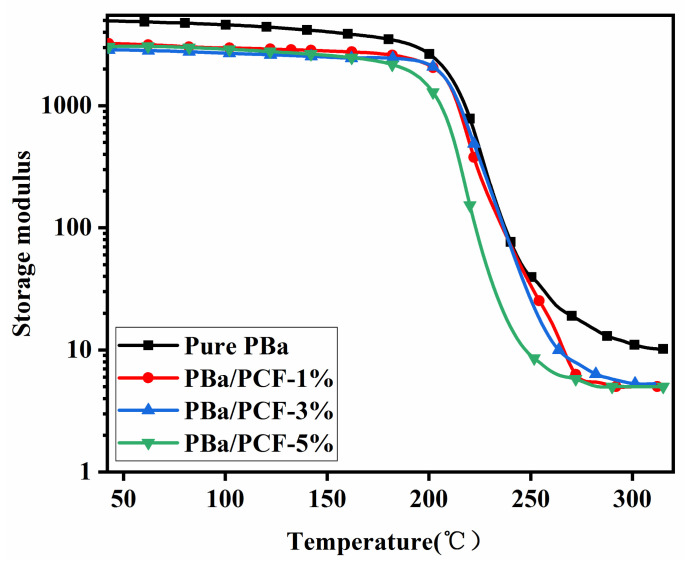
Storage modulus of PBa, PBa/PCF-1% and PBa/PCF-3% and PBa/PCF-5%.

**Figure 13:**
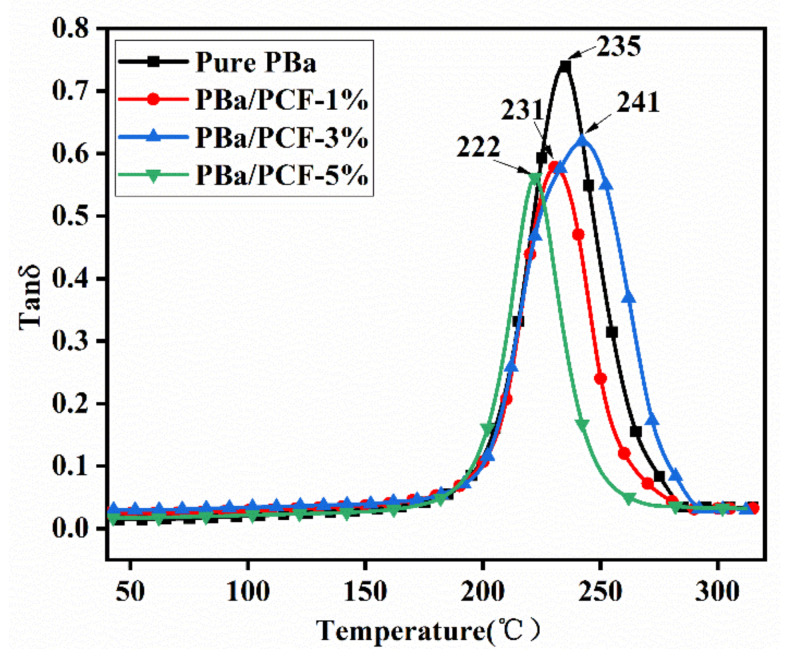
Tanδ of PBa, PBa/PCF-1%, PBa/PCF-3% and PBa/PCF-5%.

The significance of PCF in enhancing the flame retardancy of PBa was demonstrated through the results presented above. A halogen-free method to enhance the flame retardancy of polymers without sacrificing glass transition temperature (T_g_) and storage modulus is important for the purposes of academic research and industrial applications. The dynamic mechanical properties of pure PBa and its composites were measured by DMA, and the results are presented in Figure 12 and Figure 13.

The storage modulus of the PBa/PCF composites slightly decreased below T_g_ compared with that of the pure PBa (Figure 12), which was mainly due to the lower crosslink density caused by the inert additives of PCF. A similar phenomenon was observed for the T_g_ of the PBa/PCF-1% and PBa/PCF-5%. The neat PBa exhibited a T_g_ at 235 °C. The T_g_ of the PBa decreased to 231 °C with the incorporation of 1% PCF and to 222 °C with 5% PCF, which was also mainly caused by the under-curing of the PBa (Figure 13). Meanwhile, PCF is a rigid additive with abundant –OH groups, which can form hydrogen bonds with –OH in the PBa chain. The rigid backbones of PCF and the hydrogen bonding in the composite matrix can restrict the chain segment motion. The combination of the under-curing, the rigid structure of PCF and the hydrogen bonding resulted in the highest T_g_ at 241 °C and a storage modulus at 220–250 °C for the PBa/PCF-3%. In any event, the values of T_g_ were all still above 200 °C and were higher than those of traditional thermosetting resins. PCF produces no significant effects on the application temperature and working environment of PBa.

## 4. Conclusions

PBa/PCF composites were successfully fabricated and measured carefully. The effects of PCF on the thermal and flame retardancy properties of PBa were analyzed using several methods, such as DSC, TGA, CONE, and DMA. The curing temperature of PBa/PCF-5% reduced by 5 °C compared with pure PBa. The CONE and TGA analyses fully proved that the improvement in the flame retardant property of PBa was mainly due to the high weight ratio of the charred residual. The honeycomb structure of the char residue with the compact surface acted as an effective barrier for the exchange of oxygen, flame and material matrix. Furthermore, the AvHRR and mass loss values of PBa/PCF-5% decreased by 30.3% and 8.6%, respectively, compared with PBa. The T_g_ of the PBa composites was high, above 200 °C, which does not limit the application of these flame retardancy materials. This study showed that the preparation of PCF could improve many potential applications of PBa in engineering materials.

## Figures and Tables

**Table 1:** Curing characteristic parameters of Ba and Ba/PZPT systems at a heating rate of 10 °C/min.

Samples	T_i_ (°C)	T_p_ (°C)	T_f_ (°C)
Ba	215	234	280
Ba/PCF-1%	199	239	277
Ba/PCF-3%	194	236	273
Ba/PCF-5%	186	229	268

**Table 2:** Results of TGA of PBa and its composites in air and N_2_.

Sample	T_onset_ (°C)		T_max_ (°C)		Char Residue (%)			
					700 °C		800 °C	
	Air	N_2_	Air	N_2_	Air	N_2_	Air	N_2_
PBa	331	334	410	418	26	34	7	30
PBa/PCF-1%	326	323	400	404	37	35	34	34
PBa/PCF-3%	315	329	394	390	38	36	36	36
PBa/PCF-5%	302	316	380	379	39	41	37	38

**Table 3:** CONE parameters, LOI and UL-94 results of PBa and its composites.

Sample	TTI(S)	PHRR((kW/m^2^)	AvHRR(kW/m^2^)	Mass Loss(%)	LOI(%)	UL-94
Pure PBa	66	515.6	158.3	74.5%	21	NO
PBa/PCF-1%	50	406.1	152.8	75.9%	24	V-1
PBa/PCF-3%	39	397.2	163.6	68.9%	28	V-1
PBa/PCF-5%	23	212.3	110.3	65.9%	31	V0

## Data Availability

Not applicable.
